# Examination of the Fractalkine and Fractalkine Receptor Expression in Fallopian Adenocarcinoma Reveals Differences When Compared to Ovarian Carcinoma

**DOI:** 10.3390/biom5043438

**Published:** 2015-12-03

**Authors:** Hilal Gurler, Virgilia Macias, Andre A. Kajdacsy-Balla, Maria V. Barbolina

**Affiliations:** 1 Department of Biopharmaceutical Sciences, University of Illinois at Chicago, 833 South Wood Street, Chicago, IL 60612, USA; E-Mail: hgurler@uic.edu; 2 Department of Pathology, University of Illinois at Chicago, 840 South Wood Street, Chicago, IL 60612, USA; E-Mails: vmacias@uic.edu (V.M.); aballa@uic.edu (A.A.K.-B.)

**Keywords:** fractalkine, fractalkine receptor, fallopian carcinoma, ovarian carcinoma

## Abstract

Fallopian adenocarcinoma is a rare malignancy arising in the epithelium of the fallopian tube. Fallopian tube epithelium has been proposed as a tissue origin for high-grade serous ovarian carcinoma, the deadliest gynecologic malignancy. Given the commonalities in dissemination and treatment of these malignancies, we contemplated the possibility of similar patterns of gene expression underlying their progression. To reveal potential similarities or differences in the gene expression of fallopian adenocarcinoma and high-grade serous ovarian carcinoma, we tested expression of the fractalkine receptor (CX_3_CR1) and its ligand, fractalkine (CX_3_CL1), in the specimens of normal and pathologic fallopian tube using immunohistochemistry. Our data show that CX_3_CR1 is expressed in the normal, cancer adjacent normal, inflammatory, and malignant fallopian epithelium. CX_3_CL1 was expressed only by the normal and cancer adjacent normal fallopian tube epithelium; its expression was largely lost in the inflammatory and malignant fallopian epithelium. In opposite, both CX_3_CR1 and CX_3_CL1 are expressed in high-grade serous ovarian carcinoma. These findings are consistent with an idea that fallopian adenocarcinoma and high-grade serous ovarian carcinoma, although currently thought to arise from the same organ, may not share similar molecular characteristics.

## 1. Introduction

Primary fallopian tube carcinoma (PFTC) is a rare malignancy that accounts for less than 1.8% of gynecologic malignancies; the survival is quite low, at 22%–57%, and it is rarely diagnosed pre-operatively [1,2,3]. Fallopian carcinoma is thought to arise in the fallopian epithelium, and adenocarcinoma is the predominant cancer type for this malignancy [4].

Recently epithelium of the fimbriated edge of the fallopian tube gained interest as a putative site of origin for some of the cases of high-grade serous ovarian carcinoma (HGSC), the most aggressive form of epithelial ovarian carcinoma. Epithelial ovarian carcinoma (EOC), the deadliest gynecologic cancer, has long been thought to arise from the ovarian surface epithelium [5,6,7,8,9,10,11,12,13,14]. Examination of the fallopian tubes surgically removed as a prophylactic for women carriers of BRCA1/2 mutations has revealed cryptic tumors histologically resembling HGSC, and subsequent studies suggested that the majority of HGSC may arise from secretory epithelial cells on the fimbriated edge of the fallopian tube.

PFTC and EOC bear clinical and histological resemblance, although the former recurs in retroperitoneal nodes and distant sites more often than the latter [15,16,17]. Treatment of PFTC is similar to that of EOC, and it includes a total abdominal hysterectomy and omentectomy, followed by adjuvant taxane- and platinum-based chemotherapy, as well as radiotherapy [18,19,20,21,22,23]. Previous studies suggested that patients with serous PFTC are eligible for clinical trials designed for those with serous EOC [3,24]. Indeed, many currently conducted clinical trials designed for ovarian carcinoma patients also include patients with fallopian and pelvic carcinomas [25,26,27,28,29,30].

Chemokines and their receptors are important regulators of tumor progression and metastasis, including EOC [31,32]. We recently reported that the majority of both primary and metastatic high serous ovarian carcinomas express a seven transmembrane G protein-coupled receptor of chemokine family, fractalkine or CX_3_CR1, and it may play a role in increased cell migration and proliferation, as well as peritoneal adhesion to the CX_3_CL1-expressing mesothelial cells [33]. Further, we have reported that CX_3_CR1 is not expressed by the cells of normal ovarian surface epithelium (NOSE); however, its expression is gained at the earliest stages of tumorigenic transformation of NOSE [33]. It also has been found that a specific ligand of the CX_3_CR1 receptor, also known as fractalkine, or CX_3_CL1, is expressed by the ovarian carcinoma cells themselves, and a soluble form of this chemokine is present in the malignant ascites of serous ovarian carcinoma patients [33,34]. Inhibitors of CX_3_CR1 have shown promising results in the preclinical studies [35,36,37]. Hence, this molecule, given its prevalence and role in ovarian carcinoma progression, could potentially become a novel target against metastatic disease.

Expression of both CX_3_CR1 and CX_3_CL1 has been reported in the normal epithelium of the fallopian tube, where the fractalkine axis has been shown to play a role in sperm migration along the fallopian tube [34,38]. Stromal and epithelial cells were CX_3_CR1-positive [38]. However, the status of either CX_3_CR1 or CX_3_CL1 expression in fallopian tube carcinoma is unknown. Due to the proposed commonalities in the sites of origin of PFTC and HGSC, we contemplated whether these two malignancies share similar patterns of CX_3_CL1/CX_3_CR1 expression. This knowledge could aid in the effort to develop more appropriate research models and to optimize treatment of these malignancies, and it could be helpful in better understanding the etiology and development of these malignancies. In this study we tested expression of CX_3_CR1 and CX_3_CL1 in the specimens of normal and pathologic fallopian tube, with the former including specimens of normal oviduct tissue and cancer adjacent normal oviduct tissue, and the latter including specimens of fallopian tube with chronic inflammation and adenocarcinoma of the fallopian tube. These findings were analyzed in the context of previously-published data regarding expression of CX_3_CL1/CX_3_CR1 in normal epithelium of ovary and fallopian tube, as well as ovarian and fallopian carcinomas.

## 2. Results

### 2.1. Expression of CX_3_CR1 in Normal and Pathologic Fallopian Epithelium and Fallopian Carcinoma

**Figure 1:**
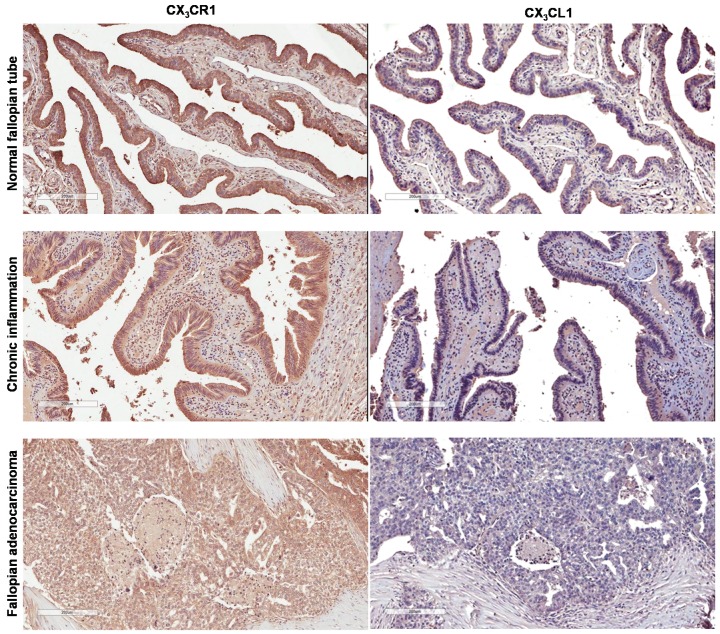
Expression of CX_3_CR1 and CX_3_CL1 in specimens of human normal fallopian tube epithelium, fallopian tube with chronic inflammation, and fallopian carcinoma. Specimens were examined for expression of CX_3_CR1 and CX_3_CL1 by immunohistochemistry. Brown—CX_3_CR1; blue—hematoxylin. Images were generated using an Aperio ScanScope digital slide scanner. Cores at positions A4 (fallopian adenocarcinoma), C4 (fallopian tube with chronic inflammation), and E9 (cancer adjacent normal oviduct tissue) were imaged (for additional information refer to Tables S1 and S2). Magnification—10×. Bar, 200 μm.

**Figure 2:**
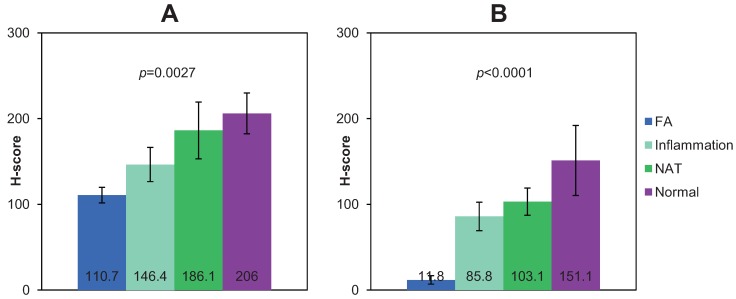
Analysis of expression of CX_3_CR1 (**A**) and CX_3_CL1 (**B**) in human specimens of fallopian adenocarcinoma (FA; *n* = 10), fallopian tube with chronic inflammation (Inflammation; *n* = 11), cancer adjacent normal oviduct tissue (NAT; *n* = 4), and normal fallopian tube epithelium (Normal; *n* = 5), as indicated. Graphs demonstrate average h-scores ± standard error. Numbers in the center of the bars indicate average h-scores. Data were statistically analyzed with one-way ANOVA.

Although expression of CX_3_CR1 in normal and pathologic ovarian surface epithelium and ovarian carcinoma as well as normal fallopian tube epithelium has been tested, the expression status of this receptor in fallopian carcinoma is not known [33,34,38]. To determine the expression of CX_3_CR1 in fallopian carcinoma, human specimens of normal, cancer adjacent to normal, inflammatory fallopian epithelium, and fallopian adenocarcinoma were immunohistochemically stained using CX_3_CR1-specific antibodies as detailed in Methods and described before [33]. The staining was evaluated only in the cells of epithelial origin, both normal and pathological. Specimens with h-scores below 100 (corresponding index scores below 1) were considered CX_3_CR1-negative, and those with h-scores above 100 (corresponding scores above 1) were considered CX_3_CR1-positive. Our data show that most of the tested specimens were CX_3_CR1-positive (Figure 1 and Table S1). Interestingly, analysis of our data shows that specimens of normal fallopian epithelium displayed the strongest intensity of the staining, as their average h-score was 206, and it was statistically significantly higher than that for specimens with chronic inflammation (average h-score = 146.4), as well as that for specimens of fallopian adenocarcinoma (average h-score = 110.7) (Figure 2). These data demonstrate that CX_3_CR1 expression is retained, albeit lowered, in the malignant epithelial cells of the fallopian tube over the course of its transformation from the normal epithelium.

### 2.2. Expression of CX_3_CL1 in Normal and Pathologic Fallopian Epithelium and Fallopian Carcinoma

CX_3_CL1 expression has been previously reported in normal and pathologic ovarian surface epithelium, ovarian carcinoma, and normal fallopian tube epithelium. To determine whether CX_3_CL1 expression is preserved in fallopian carcinoma, human specimens of normal, cancer adjacent to normal, inflammatory, fallopian epithelium, and fallopian adenocarcinoma were immunohistochemically stained using CX_3_CL1-specific antibodies as detailed in Methods. CX_3_CL1 staining in the cells of epithelial origin was evaluated using h-score as well. Specimens with h-scores below 100 were considered CX_3_CL1-negative, and those with h-scores above 100 were considered CX_3_CL1-positive. Unexpectedly, our data demonstrate that expression of CX_3_CL1 was largely lost in the specimens of fallopian carcinoma and inflammatory fallopian epithelium, as the average h-score for fallopian adenocarcinoma specimens was 11.8, and that for specimens with chronic inflammation was 85.8 (Figure 1, Figure 2 and Table S2). Although several specimens with chronic inflammation displayed h-scores above 100, most of the fallopian adenocarcinoma specimens were CX_3_CL1-negative with a few cases containing CX_3_CL1-positive cells (Table S2). Positive CX_3_CL1 expression in normal fallopian tube epithelium was reported before [34,38] and has also been confirmed by our data demonstrating the average h-score of 151.1 (Figure 1, Figure 2 and Table S2), which was statistically significantly higher than that for specimens of either fallopian carcinoma or chronic inflammation. These data strongly suggest a correlation between the tumorigenic transformation of fallopian epithelium and loss of CX_3_CL1 expression.

### 2.3. Patterns of CX_3_CL1 and CX_3_CR1 Expression Are Different between Normal Cells of Origin and Cancerous Cells in Ovarian and Fallopian Carcinomas

Both published and current data now allow analyzing the CX_3_CL1 and CX_3_CR1 expression patterns in ovarian and fallopian carcinoma and their putative cells of origin. Both ovarian surface and fallopian epithelium at the fimbriated edge were proposed as origins for high-grade serous ovarian carcinoma. According to previous reports, normal ovarian surface epithelium does not express CX_3_CR1, while the fallopian epithelium does [33,38]. CX_3_CL1 is expressed by both types of epithelium [34,38]. Both CX_3_CR1 and CX_3_CL1 are expressed by the serous ovarian carcinoma ([33,34], Table 1). Normal fallopian epithelium, which is thought to give rise to fallopian carcinoma, expresses both CX_3_CR1 and CX_3_CL1 ([34,38], Figure 1, Tables S1 and S2). However, CX_3_CL1 is expressed only by ovarian carcinoma [34], and its expression is lost in fallopian carcinoma (Table S2, Table 1 and Figure 1). Additionally, our data suggest that expression of CX_3_CR1 also declined in fallopian adenocarcinoma (average h-score = 117) as compared to normal fallopian epithelium (average h-score = 209) (Figure 2). These data may indicate that CX_3_CL1/CX_3_CR1 signaling may not play a role in fallopian adenocarcinoma, unlike ovarian carcinoma.

**Table 1:** Patterns of CX_3_CR1 and CX_3_CL1 expression in normal epithelium of ovary and fallopian tube, fallopian epithelium with chronic inflammation, ovarian carcinoma, and fallopian adenocarcinoma.

Organ Site	Normal Epithelium	Fallopian Epithelium with Chronic Inflammation	Cancer
	Expression of CX_3_CR1		
Fallopian tube	Positive ([38] and present study) Average h-score = 206	Positive (present study) Average h-score = 146.1	Positive (present study) Average h-score = 110.7
Ovary	Negative [33]		Positive [33]
	Expression of CX_3_CL1		
Fallopian tube	Positive ([34,38] and present study) Average h-score = 151.1	Negative (present study) Average h-score = 85.6	Negative (present study) Average h-score = 11.8
Ovary	Positive [34]		Positive [34]

## 3. Discussion

Malignancy collectively called “epithelial ovarian carcinoma” is a very complex disease, as it covers many histologically different subtypes. These subtypes, among which the most common are serous, endometrioid, mucinous, and clear cell, have been suggested to originate from different epithelia within the female reproductive system. Recent finding regarding cryptic tumor lesions histologically resembling high-grade serous ovarian carcinoma found within fallopian tubes led to generation of a hypothesis of fallopian origin of HGSC. According to this hypothesis HGSC originating in the fallopian tube involves ovary secondarily. Fallopian adenocarcinoma, also originating in the epithelium of the fallopian tube, disseminates within the peritoneal cavity, similar to ovarian carcinoma, and is treated using common standard of care procedures. Given such wide diversity in histotypes and cells of origin within ovarian carcinoma as well as close similarity of ovarian and fallopian carcinoma, we were interested in identifying potential common molecular pathways between ovarian and fallopian carcinomas.

We have found that CX_3_CR1 is expressed in normal and pathologic fallopian tube epithelium, as well as fallopian carcinoma, although CX_3_CR1 expression seems to significantly decline along the trajectory from normal fallopian epithelium to fallopian carcinoma. We also report that expression of CX_3_CL1 is lost in fallopian adenocarcinoma and chronically inflammatory fallopian epithelium. This may suggest that the autocrine activation of CX_3_CR1 by CX_3_CL1 in fallopian carcinoma does not take place. This is opposite from the ovarian carcinoma, where both CX_3_CR1 and CX_3_CL1 are expressed by the ovarian carcinoma cells. CX_3_CL1 is expressed by the peritoneal microenvironment and present in the malignant ascites. Hence, it may be possible that the CX_3_CL1/CX_3_CR1 interaction could potentially take place even in the absence of CX_3_CL1 expression by the fallopian carcinoma cells themselves. However, lack of available established cell culture models of the fallopian carcinoma does not allow for experimental testing of the activity of CX_3_CR1 in the processes of migration, proliferation, and adhesion it commonly supports. Additionally, it would be beneficial to test our findings using much broader sample population, including specimens from various age groups. Nevertheless, loss of CX_3_CL1 expression in the fallopian carcinoma revealed in this study, which remains expressed in ovarian carcinoma, suggests dissimilar gene expression patterns between these two malignancies. Hence, even though some high-grade ovarian carcinomas could originate from the fallopian epithelium that also gives rise to fallopian carcinoma, progression of these two malignancies may not follow the same path as far as the gene expression and, possibly, function, at least in case of the chemokine fractalkine and its receptor. Although based on the symptoms and dissemination pattern PFTC is grouped with EOC in the clinical trials, our data may suggest that the outcomes of these trials may not be the same for these two malignancies, as they may be driven by different underlying molecular mechanisms, which need to be better understood in order to design clinical trials optimized for these distinct patients cohorts.

## 4. Experimental Section

### 4.1. Materials

Tissue microarrays (TMA) containing specimens of human normal oviduct, cancer adjacent normal, hyperplasic, inflammatory, and adenocarcinoma tissues (UTE601) were obtained from US Biomax (Rockville, MD, USA). Primary antibodies produced against CX_3_CR1 and CX_3_CL1 and secondary anti-rabbit biotin-conjugated antibodies were obtained from Abcam (Cambridge, MA, USA). The Vectashield ABC kit was obtained from Vector Laboratories (Burlingame, CA, USA).

### 4.2. Immunohistochemistry

The procedure was performed as described before [33]. TMA slides were rehydrated by incubation in xylene, 100% ethanol, 95% ethanol, 70% ethanol, and phosphate-buffered saline, pH 7.4, for 5 min each. Peroxidase activity was inhibited with H_2_O_2_. Antigen retrieval was performed by a 15 min incubation in 1 mM ethylene diamine tetra acetic acid (EDTA; pH 8.0) at 95 °C. Prior to primary antibody staining, non-specific binding was blocked by incubation with 10% goat serum for 1 h. The primary CX_3_CR1-specific antibody was used at 1:50 dilution for 1 h at room temperature (RT). The primary CX_3_CL1-specific antibody was used at 1 μg/mL overnight at 4 °C. The biotin-conjugated goat anti-rabbit secondary antibody was used at a 1:200 dilution for 30 min at RT. The Vectashield ABC kit was used as directed by the manufacturer, and tissues were incubated for 45 min at RT. The DAB reagent was prepared as instructed by the manufacturer and applied to tissues on TMA slides for 2–10 min until brown color developed. TMAs were stained with hematoxylin, dehydrated in 50%, 70%, 95%, and 100% ethanol, and mounted with Permount. Staining was scored based on the intensity and percentage of positive cells in the entire section; two sections per case were analyzed. Intensity of staining was “0” for negative samples, “1” for weakly positive samples, “2” for moderately positive samples, and “3” for highly positive samples. H-score (range, 0–300) was calculated by multiplying the percentage by the intensity as described before [39].

## 5. Conclusions

In this report we evaluated expression of a chemokine fractalkine (CX_3_CL1) and its receptor CX_3_CR1 in the specimens of normal and pathological fallopian tube. Our data suggest that, unlike in the ovarian carcinoma, autocrine activation of CX_3_CR1 by CX_3_CL1 in fallopian carcinoma cells may not occur. This may suggest that progression of fallopian adenocarcinoma and ovarian carcinoma is not supported by the same mechanisms.

## Supplementary Files

Supplementary File 1
