# Supplementary material for: Examination of the Fractalkine and Fractalkine Receptor Expression in Fallopian Adenocarcinoma Reveals Differences When Compared to Ovarian Carcinoma
Source: Biomolecules. 2015 Dec 3;5(4):3438–47. doi: 10.3390/biom5043438 (PMC4693285; doi:10.3390/biom5043438)
Supplement: Supplementary File 1 [file biomolecules-05-03438-s001.pdf]

Supplementary Materials

Table S1. Expression of CX3CR1 in human specimens or normal and diseased fallopian tube.

Position	Case [#]	Sex	Age	Organ	Pathology [*,#]	Grade	Type	0+	1+	2+	3+	Index Score	H-Score	Comments
A1	1	F	47	FT [&]	Adenocarcinoma	2	Malignant	1%	84%	15%		1.14	114	
A2	1	F	47	FT	Adenocarcinoma	2	Malignant	1%	89%	10%		1.09	109	
A3	2	F	46	FT	Adenocarcinoma	3	Malignant		85%	15%		1.15	115	
A4	2	F	46	FT	Adenocarcinoma	3	Malignant		80%	20%		1.2	120	
A5	3	F	43	FT	Adenocarcinoma	2–3	Malignant	1%	4%	95%		1.94	194	
A6	3	F	43	FT	Adenocarcinoma	2–3	Malignant		25%	25%	50%	2.25	225	most missing
A7	4	F	52	FT	Adenocarcinoma	3	Malignant	25%	65%	10%		0.85	85	
A8	4	F	52	FT	Adenocarcinoma	3	Malignant	10%	75%	15%		1.05	105	
A9	5	F	53	FT	Adenocarcinoma	3	Malignant	35%	45%	20%		0.85	85	
A10	5	F	53	FT	Adenocarcinoma	3	Malignant	15%	70%	15%		1	100	
B1	6	F	60	FT	Adenocarcinoma	3	Malignant	1%	84%	15%		1.14	114	
B2	6	F	60	FT	Adenocarcinoma	3	Malignant	10%	80%	10%		1	100	
B3	7	F	40	FT	Adenocarcinoma	3	Malignant	10%	85%	5%		0.95	95	
B4	7	F	40	FT	Adenocarcinoma	3	Malignant	5%	90%	5%		1	100	
B5	8	F	57	FT	Adenocarcinoma	3	Malignant	1%	79%	20%		1.19	119	
B6	8	F	57	FT	Adenocarcinoma	3	Malignant	10%	90%				90	most missing
B7	9	F	51	FT	Adenocarcinoma	3	Malignant		85%	15%		1.15	115	
B8	9	F	51	FT	Adenocarcinoma	3	Malignant		80%	20%		1.2	120	
B9	10	F	53	FT	Adenocarcinoma	3	Malignant	35%	15%	50%		1.15	115	
B10	10	F	53	FT	Adenocarcinoma	3	Malignant	35%	40%	10%	15%	1.05	105	
C1	11	F	41	FT	Chronic inflammation	-	Inflammation					n/a	n/a	no epithelium
C2	11	F	41	FT	Chronic inflammation	-	Inflammation					n/a	n/a	no epithelium
C3	12	F	32	FT	Chronic inflammation	-	Inflammation	15%	65%	15%	5%	1.1	110	
C4	12	F	32	FT	Chronic inflammation	-	Inflammation	5%	30%	45%	20%	1.8	180	
C5	13	F	46	FT	Chronic inflammation	-	Inflammation	1%	24%	45%	30%	2.04	204	
C6	13	F	46	FT	Chronic inflammation	-	Inflammation					n/a	n/a	no epithelium
C7	14	F	34	FT	Chronic inflammation	-	Inflammation					n/a	n/a	no epithelium

Table S1. *Cont.*

Position	Case #	Sex	Age	Organ	Pathology *, #	Grade	Type	0+	1+	2+	3+	Index Score	H-Score	Comments
C8	14	F	34	FT	Chronic inflammation	-	Inflammation	1%	4%	80%	15%	2.09	209	
C9	15	F	34	FT	Chronic inflammation	-	Inflammation	5%	10%	65%	20%	2	200	
C10	15	F	34	FT	Chronic inflammation	-	Inflammation	5%	5%	55%	35%	2.2	220	
D1	16	F	53	FT	Chronic inflammation	-	Inflammation					n/a	n/a	no epithelium
D2	16	F	53	FT	Chronic inflammation	-	Inflammation					n/a	n/a	no epithelium
D3	17	F	49	FT	Chronic inflammation	-	Inflammation	20%	75%	5%		0.85	85	
D4	17	F	49	FT	Chronic inflammation	-	Inflammation	15%	70%	10%	5%	1.05	105	
D5	18	F	49	FT	Chronic inflammation	-	Inflammation					n/a	n/a	no epithelium
D6	18	F	49	FT	Chronic inflammation	-	Inflammation	5%	20%	75%		1.7	170	
D7	19	F	22	FT	Chronic inflammation	-	Inflammation	2%	40%	53%	5%	1.61	161	
D8	19	F	22	FT	Chronic inflammation	-	Inflammation	2%	30%	53%	15%	1.81	181	
D9	20	F	29	FT	Chronic inflammation	-	Inflammation	2%	13%	45%	40%	2.23	223	
D10	20	F	29	FT	Chronic inflammation	-	Inflammation		5%	40%	55%	2.5	250	
E1	21	F	27	FT	Chronic inflammation	-	Hyperplasia	95%	5%			0.05	5	
E2	21	F	27	FT	Chronic inflammation	-	Hyperplasia	60%	40%			0.4	40	
E3	22	F	56	FT	Cancer adjacent normal oviductal tissue	-	NAT	5%	25%	45%	25%	1.9	190	
E4	22	F	56	FT	Cancer adjacent normal oviductal tissue	-	NAT		25%	55%	20%	1.95	195	
E5	23	F	34	FT	Cancer adjacent normal oviductal tissue	-	NAT			25%	75%	2.75	275	
E6	23	F	34	FT	Cancer adjacent normal oviductal tissue	-	NAT	1%	10%	54%	35%	2.23	223	
E7	24	F	26	FT	Cancer adjacent normal oviductal tissue	-	NAT					n/a	n/a	no epithelium
E8	24	F	26	FT	Cancer adjacent normal oviductal tissue	-	NAT					n/a	n/a	no epithelium

Table S1. *Cont.*

Position	Case #	Sex	Age	Organ	Pathology *,#	Grade	Type	0+	1+	2+	3+	Index Score	H-Score	Comments
E9	25	F	47	FT	Cancer adjacent normal oviductal tissue	-	NAT	5%	10%	75%	10%	1.9	190	
E10	25	F	47	FT	Cancer adjacent normal oviductal tissue	-	NAT		10%	50%	40%	2.3	230	
F1	26	F	21	FT	Normal oviductal tissue	-	Normal		5%	75%	20%	1.85	185	
F2	26	F	21	FT	Normal oviductal tissue	-	Normal			55%	45%	2.45	245	
F3	27	F	15	FT	Normal oviductal tissue	-	Normal		5%	55%	40%	2.35	235	
F4	27	F	15	FT	Normal oviductal tissue	-	Normal			55%	45%	2.45	245	
F5	28	F	18	FT	Normal oviductal tissue	-	Normal			45%	55%	2.55	255	
F6	28	F	18	FT	Normal oviductal tissue	-	Normal			75%	25%	2.25	225	
F7	29	F	21	FT	Normal oviductal tissue	-	Normal			95%	5%	2.05	205	
F8	29	F	21	FT	Normal oviductal tissue	-	Normal		10%	50%	40%	2.3	230	
F9	30	F	21	FT	Normal oviductal tissue	-	Normal		5%	55%	40%	2.35	235%	
F10	30	F	21	FT	Normal oviductal tissue	-	Normal					n/a	n/a	no epithelium

* Information obtained from the manufacturer (US Biomax; Rockville, MD, USA) as it pertains to the tissue microarray UTE601. # Diagnosis was confirmed by the gynecologic pathologist Andre A. Kajdacsy-Balla. & FT designates fallopian tube. Index score is calculated by dividing h-score by 100.

Table S2. Expression of CX3CL1 in human specimens or normal and diseased fallopian tube.

Position	Case #	Sex	Age	Organ	Pathology *,#	Grade	Type	0+	1+	2+	3+	Index Score	H-Score	Comments
A1	1	F	47	FT &	Adenocarcinoma	2	Malignant	90%	10%			0.1	10	
A2	1	F	47	FT	Adenocarcinoma	2	Malignant	95%	5%			0.05	5	
A3	2	F	46	FT	Adenocarcinoma	3	Malignant	85%	15%			0.15	15	
A4	2	F	46	FT	Adenocarcinoma	3	Malignant	95%	5%			0.05	5	
A5	3	F	43	FT	Adenocarcinoma	2–3	Malignant	95%	5%			0.05	5	
A6	3	F	43	FT	Adenocarcinoma	2–3	Malignant	100%				0	0	
A7	4	F	52	FT	Adenocarcinoma	3	Malignant	100%				0	0	
A8	4	F	52	FT	Adenocarcinoma	3	Malignant	100%				0	0	

Table S2. *Cont.*

Position	Case #	Sex	Age	Organ	Pathology *,#	Grade	Type	0+	1+	2+	3+	Index Score	H-Score	Comments
A9	5	F	53	FT	Adenocarcinoma	3	Malignant	95%		5%		0.1	10	
A10	5	F	53	FT	Adenocarcinoma	3	Malignant	94%	5%		1%	0.08	8	
B1	6	F	60	FT	Adenocarcinoma	3	Malignant	85%	15%			0.15	15	
B2	6	F	60	FT	Adenocarcinoma	3	Malignant	95%	5%			0.05	5	
B3	7	F	40	FT	Adenocarcinoma	3	Malignant	95%	5%			0.05	5	
B4	7	F	40	FT	Adenocarcinoma	3	Malignant	95%	5%			0.05	5	
B5	8	F	57	FT	Adenocarcinoma	3	Malignant	100%				0	0	
B6	8	F	57	FT	Adenocarcinoma	3	Malignant	100%				0	0	
B7	9	F	51	FT	Adenocarcinoma	3	Malignant	100%				0	0	
B8	9	F	51	FT	Adenocarcinoma	3	Malignant	95%	5%			0.05	5	
B9	10	F	53	FT	Adenocarcinoma	3	Malignant	25%	75%			0.75	75	
B10	10	F	53	FT	Adenocarcinoma	3	Malignant	20%	80%			0.8	80	
C1	11	F	41	FT	Chronic inflammation	-	Inflammation					n/a	n/a	no epithelium
C2	11	F	41	FT	Chronic inflammation	-	Inflammation					n/a	n/a	no epithelium
C3	12	F	32	FT	Chronic inflammation	-	Inflammation	5%	90%		5%	1.05	105	
C4	12	F	32	FT	Chronic inflammation	-	Inflammation	5%	90%		5%	1.05	105	
C5	13	F	46	FT	Chronic inflammation	-	Inflammation	1%	10%	10%	79%	2.67	267	
C6	13	F	46	FT	Chronic inflammation	-	Inflammation					n/a	n/a	no epithelium
C7	14	F	34	FT	Chronic inflammation	-	Inflammation					n/a	n/a	no epithelium
C8	14	F	34	FT	Chronic inflammation	-	Inflammation		99%		1%	1.02	102	
C9	15	F	34	FT	Chronic inflammation	-	Inflammation	15%	80%	5%		0.9	90	
C10	15	F	34	FT	Chronic inflammation	-	Inflammation	5%	80%	10%	5%	1.15	115	
D1	16	F	53	FT	Chronic inflammation	-	Inflammation					n/a	n/a	no epithelium
D2	16	F	53	FT	Chronic inflammation	-	Inflammation					n/a	n/a	no epithelium
D3	17	F	49	FT	Chronic inflammation	-	Inflammation	20%	40%	40%		1.2	120	
D4	17	F	49	FT	Chronic inflammation	-	Inflammation	40%	5%	50%	5%	1.2	120	
D5	18	F	49	FT	Chronic inflammation	-	Inflammation		95%	5%		1.05	105	
D6	18	F	49	FT	Chronic inflammation	-	Inflammation		95%	5%		1.05	105	

Table S2. *Cont.*

Position	Case #	Sex	Age	Organ	Pathology *, #	Grade	Type	0+	1+	2+	3+	Index Score	H-Score	Comments
D7	19	F	22	FT	Chronic inflammation	-	Inflammation	15%	75%	10%		0.95	95	
D8	19	F	22	FT	Chronic inflammation	-	Inflammation	5%	70%	20%	5%	1.25	125	
D9	20	F	29	FT	Chronic inflammation	-	Inflammation	100%				0	0	
D10	20	F	29	FT	Chronic inflammation	-	Inflammation	100%				0	0	
E1	21	F	27	FT	Chronic inflammation	-	Hyperplasia	100%				0	0	
E2	21	F	27	FT	Chronic inflammation	-	Hyperplasia	70%	15%	10%	5%	0.5	5	
E3	22	F	56	FT	Cancer adjacent normal oviductal tissue	-	NAT		60%	25%	15%	1.55	155	
E4	22	F	56	FT	Cancer adjacent normal oviductal tissue	-	NAT	5%	80%	5%	10%	1.2	120	
E5	23	F	34	FT	Cancer adjacent normal oviductal tissue	-	NAT	5%	75%	10%	10%	1.25	125	
E6	23	F	34	FT	Cancer adjacent normal oviductal tissue	-	NAT	5%	85%	10%		1.05	105	
E7	24	F	26	FT	Cancer adjacent normal oviductal tissue	-	NAT					n/a	n/a	no epithelium
E8	24	F	26	FT	Cancer adjacent normal oviductal tissue	-	NAT	5%	85%	10%		1.05	105	
E9	25	F	47	FT	Cancer adjacent normal oviductal tissue	-	NAT		85%	15%		1.15	115	
E10	25	F	47	FT	Cancer adjacent normal oviductal tissue	-	NAT	5%	90%	5%		1	100	
F1	26	F	21	FT	Normal oviductal tissue	-	Normal					n/a	n/a	missing
F2	26	F	21	FT	Normal oviductal tissue	-	Normal					n/a	n/a	missing
F3	27	F	15	FT	Normal oviductal tissue	-	Normal		5%	25%	70%	2.65	265	
F4	27	F	15	FT	Normal oviductal tissue	-	Normal	5%	10%	20%	65%	2.45	245	
F5	28	F	18	FT	Normal oviductal tissue	-	Normal		50%	20%	30%	1.8	180	
F6	28	F	18	FT	Normal oviductal tissue	-	Normal	10%	75%	10%	5%	1.1	110	
F7	29	F	21	FT	Normal oviductal tissue	-	Normal	5%	45%	45%	5%	1.5	150	
F8	29	F	21	FT	Normal oviductal tissue	-	Normal		80%	15%	5%	1.25	125	
F9	30	F	21	FT	Normal oviductal tissue	-	Normal	5%	95%			0.95	95	
F10	30	F	21	FT	Normal oviductal tissue	-	Normal		10%	90%		1.9	190	

* Information obtained from the manufacturer (US Biomax; Rockville, MD, USA) as it pertains to the tissue microarray UTE601. # Diagnosis was confirmed by the gynecologic pathologist Andre A. Kajdacsy-Balla. & FT designates fallopian tube. Index score is calculated by dividing h-score by 100.
